# Altered neurobehavioral white matter integrity in preterm children: A confounding-controlled analysis using the adolescent brain and cognitive development (ABCD) study

**DOI:** 10.1016/j.neuroimage.2025.121600

**Published:** 2025-11-21

**Authors:** Hailong Li, Yuwen Hung, Junqi Wang, Nicole Rudberg, Nehal A. Parikh, Lili He

**Affiliations:** a Imaging Research Center, Department of Radiology, Cincinnati Children’s Hospital Medical Center, Cincinnati, OH, USA; b Neurodevelopmental Disorders Prevention Center, Perinatal Institute, Cincinnati Children’s Hospital Medical Center, Cincinnati, OH, USA; c Department of Radiology, University of Cincinnati College of Medicine, Cincinnati, OH, USA; d Athinoula A. Martinos Center for Biomedical Imaging, Harvard-MIT, Cambridge, MA, USA; e Department of Pediatrics, University of Cincinnati College of Medicine, Cincinnati, OH, USA; f Department of Computer Science, Department of Biomedical Engineering, Department of Biomedical Informatics, University of Cincinnati, Cincinnati, OH, USA

**Keywords:** Preterm children, Diffusion tensor imaging, White matter integrity

## Abstract

**Introduction:**

Children born preterm face elevated risks of atypical brain development and neurodevelopmental difficulties. However, little is known about childhood outcomes specifically associated with premature birth that are unconfounded by medical complications. This study takes a systematic approach to examine neural and behavioral outcomes in non-medically complex preterm children. The aim is to identify unconfounded neurobehavioral biomarkers and mechanisms that contribute to childhood vulnerability following premature birth, ultimately informing the development of effective interventions to mitigate adverse outcomes in this population.

**Methods:**

This study leverages the largest publicly available prospective dataset on child brain health in the U.S.—the Adolescent Brain and Cognitive Development (ABCD) Study—using a case-control design. Applying rigorous, systematic confounding control procedures, the study includes 612 children aged 9–11 who have been free of medical and developmental complications since birth. The cohort comprises 306 children born preterm and 306 full-term children matched for age, sex, and socioeconomic status. A comprehensive range of neurocognitive outcomes is examined in relation to the integrity of brain connectomes, as measured by diffusion tensor imaging (DTI).

**Results:**

Preterm children and full-term control children are significantly differentiated by altered microstructural and axonal integrity, in major frontal-limbic tracts in the whole brain (*p* < .05). In particular, the strength of structural connectivity in the anterior thalamic radiation, which connects the frontal lobe and the thalamic sensory relay circuit, shows altered brain-behavioral regulatory relationship with the performance on the attention and processing speed task (*p* < .05).

**Conclusion:**

This secondary analysis of the ABCD Study identified unconfounded neurobehavioral risk biomarkers associated with premature birth, along with underlying neurobiological and cognitive mechanisms. Children born preterm demonstrated reduced neurobehavioral white matter integrity within the frontal-limbic connectome, particularly in tasks requiring sustained attention and processing speed. This diminished adaptability places them at elevated risk for developing related neurodevelopmental difficulties. These findings highlight the urgent need for routine screening and preventive neuro-rehabilitative interventions—such as attention-focused and sensory-feedback-based training—for preterm-born populations.

## Introduction

Children born preterm, a condition that impacts about 10 % of all live birth globally (i.e., born < 37 weeks), (Chawanpaiboon et al., 2019) are at increased risk of impaired neurobehavioral development. (Ball et al., 2015; Bhutta et al., 2002) This creates pressing public health challenges, as preterm-born children have been found to exhibit increased rates of developing perceptual and motor related impairments, (Allotey et al., 2018) learning difficulties, (Carmo et al., 2021) academic challenges as well as increased incidents of attention deficit/hyperactivity disorder. (Allotey et al., 2018; Sucksdorff et al., 2015) Previous preterm research that studied disabilities and adverse neurodevelopmental outcomes has mostly focused on cohorts born with certain additional birth complications, such as born very preterm, with very low birth weights, with neurological or other medical complications. (Serenius et al., 2013; Anderson and Doyle, 2003; Woodward et al., 2006) Little is known, however, about the unconfounded nature of childhood neurobehavioral impacts linked to premature birth—impacts and risks that are not observable at birth or attributable to other medical complications, but may leave the preterm-born child susceptible to adverse environmental conditions later on. The lack of such knowledge about the unconfounded childhood impacts of premature birth has precluded the development of effective interventions that can prevent negative prospects and ensure positive outcomes for preterm cohorts.

Confounding issues have limited the ability to draw accurate conclusions about long-term development of prematurity. There are major confounding issues that remain unresolved in current literature. First, while there is a relatively direct causal relationship between a given perinatal condition and early-life outcome, there exist difficulties in implementing systematic quality control of confounding factors without significant data loss in studying children who were born preterm years ago. (Aylward, 2002a; Ment et al., 2003) Key confounding factors include developmental, medical, and mental health history, in addition to the commonly considered sociodemographic factors, which all contribute to the childhood outcomes. Second, there exist diverse behavior reports on preterm children, which is attributed to varied and narrow ranges of cognitive outcome measures used among studies. Broader spectrums of cognitive-behavioral measures should be considered to make findings not only comparable across studies but also generalizable to the full-range daily life demands in childhood. (Bhutta et al., 2002; Aylward, 2002a, 2002b)

To resolve current major methodological issues, this study implements a systematic confounding control approach using the largest prospective study of brain development and child health in the U.S.—the Adolescent Brain and Cognitive Development (ABCD) study. (Barch et al., 2018) We implement a rigorous systematic confounding control approach by assessing the integrity of the childhood brain connectomes and full-range cognitive outcomes of school-age children who were born preterm without postnatal medical and since-birth developmental complications. The aim of this study is to identify unconfounded neurobehavioral abnormalities that underline childhood susceptibilities as the result of premature birth in a non-medically complex preterm cohort. The large sample size of the ABCD dataset and its full-range records of at-birth and since-birth medical and mental health histories enable us to employ rigorous systematic procedures to rule out critical confounding factors unaddressed in previous research. We assess a full range of cognitive measures involving distinct cognitive functionality spanning from early to late information processing stages, including attention and processing speed, inhibitory control, executive function, and working memory, as well as language function.

Moreover, we investigate the integrity of the brain connectomes in the study eligible children (ages 9–11 years, free from neurological, mental, and other medical conditions since birth, detailed in Methods) by examining their diffusion tensor imaging (DTI) records. DTI measures (e.g., fractional anisotropy (FA)) can indicate the strength and directionality of microstructural connectivity in the brain’s white matter tracts and provide information about axonal health and myelin sheath integrity. (Beaulieu, 2002; Hutchinson et al., 2018) Abnormalities in white matter microstructures, as identified by DTI measures, have been found to be indicative of neurodevelopmental outcomes in preterm populations. (Woodward et al., 2006; He and Parikh, 2013; Bugada et al., 2021) Existing findings in preterm populations have been highly variable, (Dimitrova et al., 2020) which showed diffuse patterns of impairments that reflect a non-specific, below-maturational level in general. (He and Parikh, 2015; Kline et al., 2021)

To our knowledge, the current study is the first one to implement a systematic quality control method to identify the undiscovered childhood neural-behavioral risk biomarkers linked to premature birth, using the largest available developmental dataset (the ABCD Study). The results of the study provide new knowledge about the unconfounded nature of childhood impacts following premature birth. The results can be used to design effective interventions that can forestall negative neurobehavioral developments for preterm cohorts, including those non-medically complex but at-risk preterm cohorts that have been previously overlooked.

## Materials & methods

### Participant inclusion/exclusion and quality control procedure

**Fig. 1.**
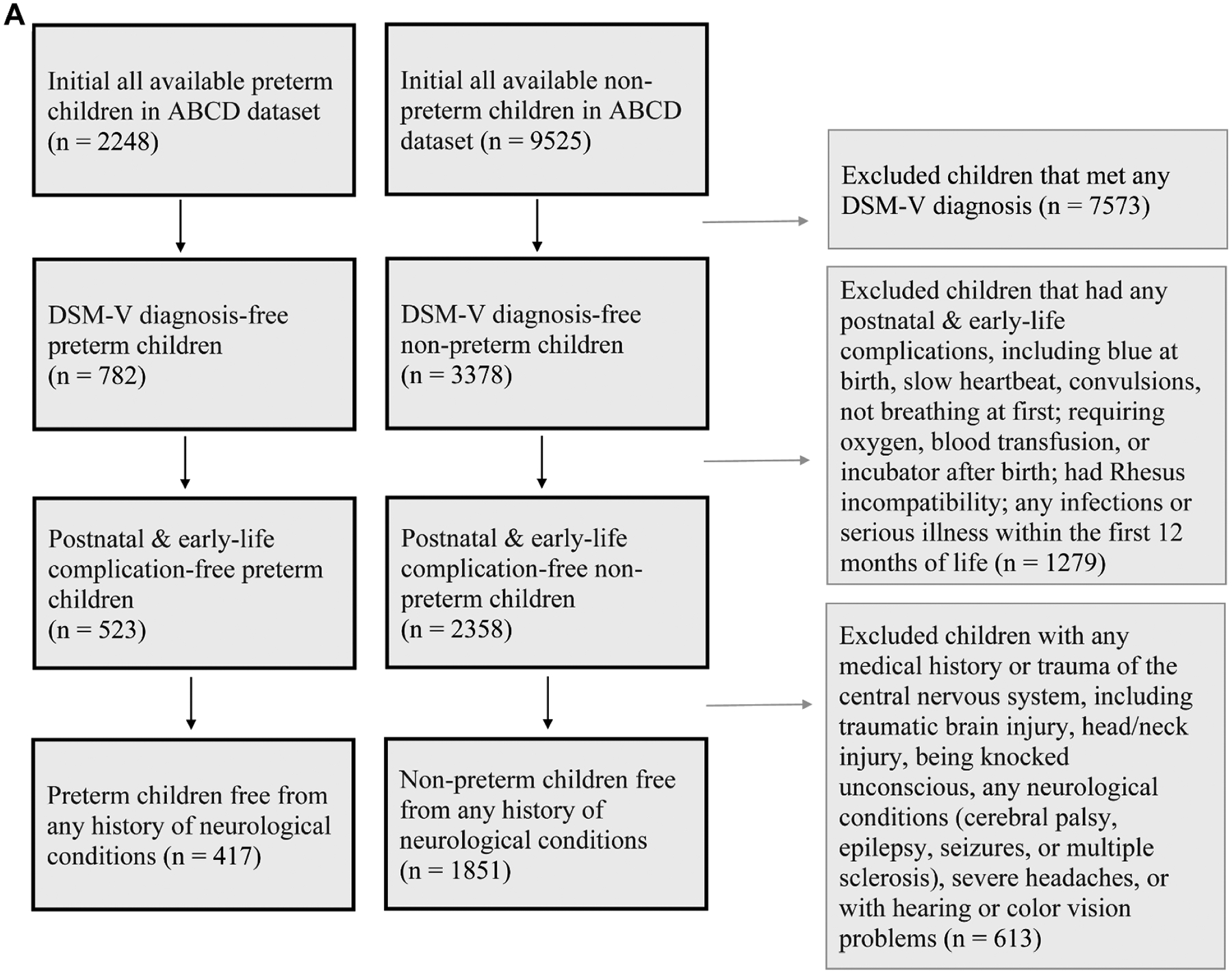

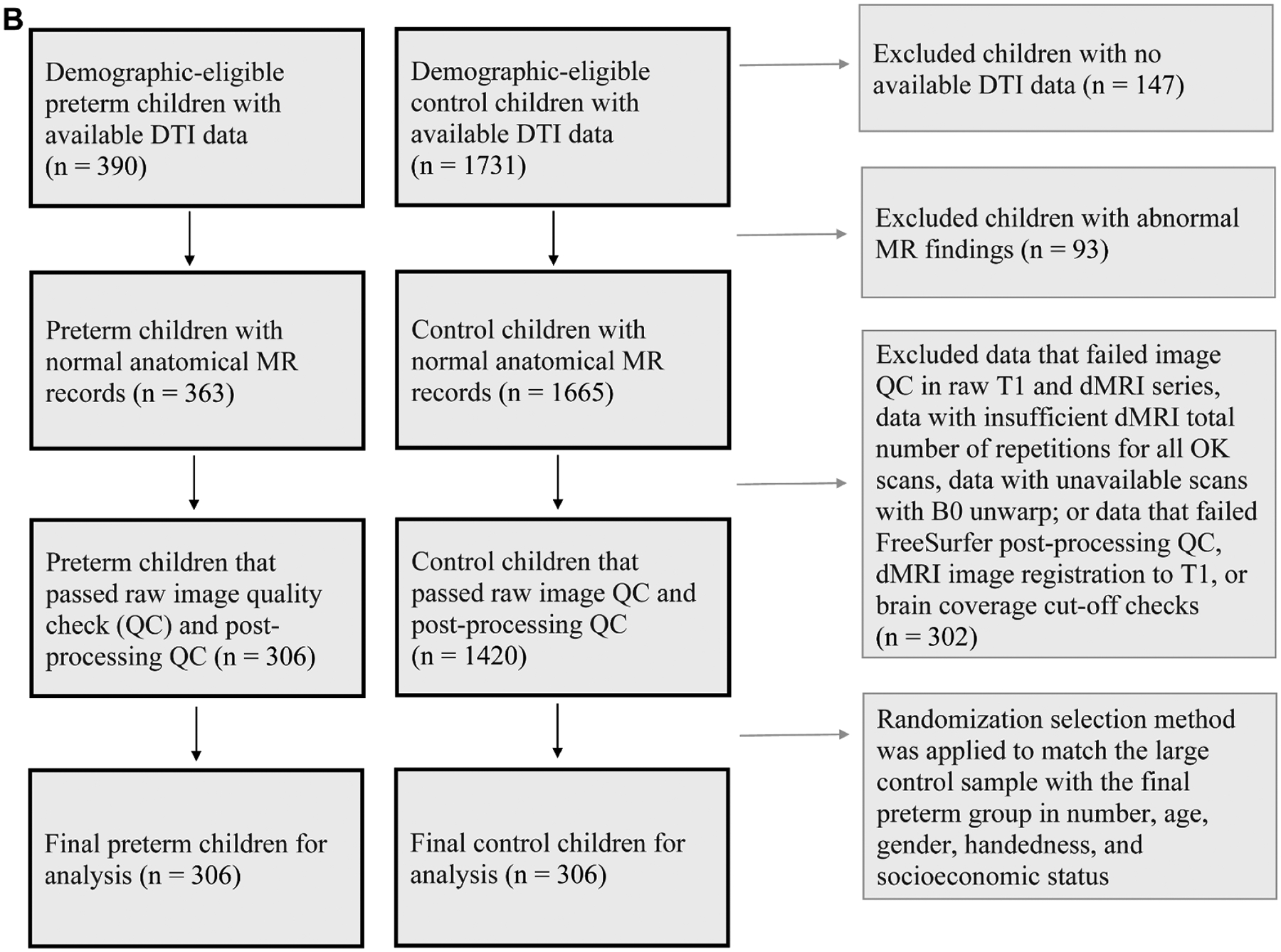
Quality control procedures using the ABCD database for identification of study-eligible, non-medically compromised children born preterm and matched full-term control children.

The ABCD study participants were recruited from a total of 21 educational sites across the United States, consisting of a baseline cohort of nearly 12,000 children 9–11 years old (and their parents/guardians) that reflect the sociodemographic variation of the US population and are being followed up with annual lab-based assessments, including Magnetic Resonance Imaging (MRI). A total of 2208 children born preterm and 9525 children born full-term were identified from the baseline recruitment in the ABCD 2020 Data Release 3.0. In these participants, we excluded children with a history of any DSM-V diagnosis of psychiatric disorders (e.g., Autism Spectrum Disorder, Attention-Deficit/Hyperactivity Disorder); with presence of any brain injury, structural lesion, or other clinically significant incidental finding on the anatomical MRI scans upon radiological review, with any postnatal and early-life medical complications, or with a history of head trauma or neurological conditions that required medical care, as detailed in Fig. 1A and the Supplementary Methods.

### Neuroimaging and quality control process

The above demographic-eligible participants were entered into the imaging data quality control process, which includes examinations of the raw and the pre-processed imaging data, as detailed in Fig. 1B and the Supplementary Methods, along with the imaging acquisition. Specifically, we performed one-to-one propensity score matching (PSM) (Austin, 2011) between the preterm and control groups without replacement. A logistic regression model was used to predict group membership (preterm vs. control) based on key demographic and clinical covariates, including age, sex, two socioeconomic factors (primary caregiver’s educational years and the total family annual income), and handedness. From the model, a propensity score was calculated for each participant. Each preterm subject was then matched to a control subject with the closest propensity score, using a caliper width of 0.2 standard deviations. This process resulted in a final matched cohort of 306 preterm subjects and 306 control subjects (Fig. 1B; Table 1). Post-matching diagnostics confirmed that the propensity score matching effectively balanced all included covariates between the two groups. (Fig. 1B; Table 1). After PSM matching, the age at scan and at neurocognitive assessment for the preterm group is 10.03 ± 0.60 (mean ± standard deviation) years, and for the term control group is 10.03 ± 0.55. Analyzed DTI measures include fractional anisotropy (FA), mean diffusivity (MD), axial diffusivity (AD), and radial diffusivity (RD). FA reflects the degree of directional water diffusion and is generally interpreted as an index of overall white matter integrity. MD represents the overall magnitude of water diffusion and is sensitive to tissue density and cellularity. AD indexes diffusion along the primary axis of the fiber bundle and has been associated with axonal integrity. RD reflects diffusion perpendicular to axonal fibers and is often interpreted as a marker of myelin integrity. These 4 diffusion measures were extracted from a total of 30 white matter regions consisting of 15 major cortico-cortical, cortico-subcortical, and subcortical tracts throughout the whole brain, as detailed in the Supplementary Methods. A global FA value (mean FA value of the whole brain) is used for each participant as a covariate to control for individual baseline connectivity variation in all analyses.

### Neurocognitive measurement

We use structured multi-dimensional cognitive tests from the NIH Toolbox provided in the ABCD dataset including: (1) NIH Toolbox Pattern Comparison Processing Speed – measuring attention and information processing speed; (2) NIH Toolbox Flanker Inhibitory Control And Attention Test – measuring inhibition and cognitive control (executive function); (3) NIH Toolbox Dimensional Change Card Sort Test – measuring cognitive flexibility (executive function); (4) NIH Toolbox List Sorting Working Memory Test – measuring visual and auditory working memory capacity; (5) NIH Toolbox Picture Vocabulary Test – measuring language ability and vocabulary knowledge; (6) NIH Toolbox Picture Sequence Memory Test – measuring episodic memory; (7) NIH Toolbox Oral Reading Recognition – measuring reading decoding skills and crystalized abilities. Detailed information about these tests can be found in the Supplementary Methods and the NIH Toolbox website: https://www.nihtoolbox.org/. Raw scores for each test were obtained and converted to age-adjusted standard scores; the latter were used for all analyses.

### Data analysis

To determine the relationship between Group and FA in any white matter regions throughout the whole brain (predictor variables), logistic regression analysis was carried out to identify significant brain regions able to distinguish the Preterm vs. Control (Group) variable while holding all other variables constant (including the global FA covariate). Stepwise forward likelihood ratio selection method was used to identify the most significant predictors among all brain regions. Multiple comparisons were corrected using the Bonferroni method. The final regression model was tested using all surviving predictors. For each significant tract, group comparison tests were conducted using non-parametric (Quade’s) one-way ANCOVA (Quade, 1967) to identify any differences between groups as a supplementary assessment. The MD, AD, and RD metrics were examined independently as secondary analyses in each identified brain region to supplement FA findings and determine the underlying neurophysiological mechanisms. Furthermore, brain-behavior relationships were investigated for each significant neural region in relation to the functionality of every cognitive dimension. Within-group brain-behavior correlations were first carried out using the FA and the (age-corrected) standard scores of each cognitive test using Pearson’s Correlation tests. Then, for any significant within-group finding, group comparisons were conducted to determine whether the brain-behavior relationship differed between groups. Specifically, two-way ANCOVA (non-parametric) was carried out to test any interaction effect between Group and specific cognitive test performance that impacts the brain’s microstructural integrity.

## Results

**Fig. 2.**
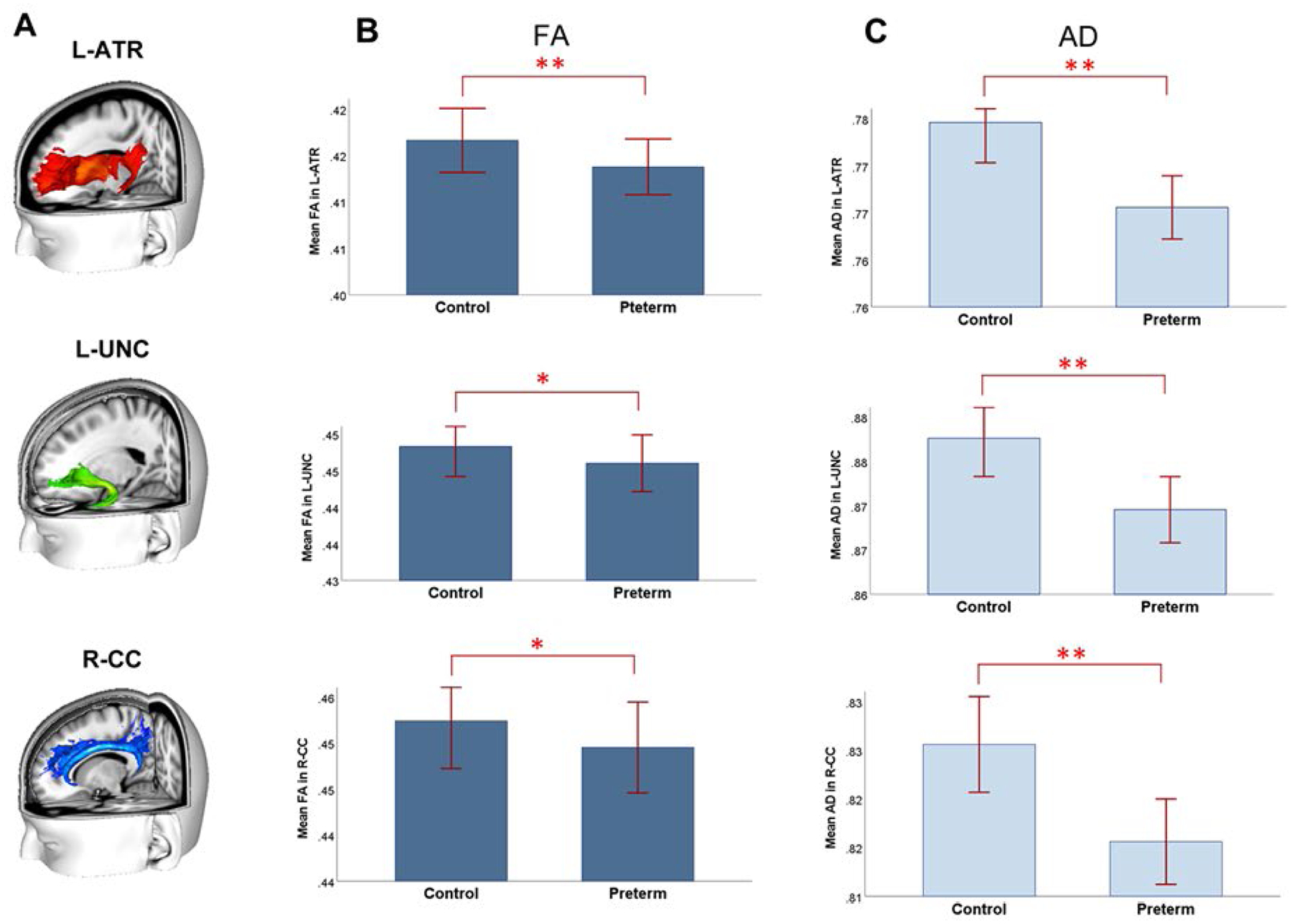
Preterm children are differentiated from the non-preterm control children by connectivity strength in the frontal-subcortical white matter tracts among all brain tracts. (A) The three significant white matter tracts are 3D-rendered (in different colors) using the JHU white matter tractography atlas, overlaid in the 3D-rendered standard T1 brain template (MNI 152 1 mm image). l-ATR = Left anterior thalamic radiation; l-UNC = Left uncinate fasciculus; R-CC = Right cingulate-cingulum tract. (B) FA in these identified tracts shows a pattern of Preterm < Control group differences, especially in the ATR. ** < 0.05, * <0.07. (C) The AD scores in these tracts show a pattern of Preterm > Control differences. Note that these group differences are not influenced by the individual global FA differences (baseline). Error bars represent ± 2 standard errors.

Logistic regression analysis results show that the likelihood of each individual child being preterm (probability = 1) or control (probability = 0) is significantly differentiated by FA in the left anterior thalamic radiation (L-ATR), left uncinate fasciculus (L-UNC), and the right cingulate-cingulum (R-CC), while holding constant all other brain regions’ connectivity levels (including the global FA value) (*P* < .05, corrected). The unstandardized beta (*B*) weight parameters in the final model with the surviving significant tracts are: l-ATR: *B* = −11.593, *SE* = 4.287, *Wald* = 7.313, *P* < .007; l-UNC: *B* = −10.304, *SE* = 3.789, *Wald* = 7.394, *P* < .007; R-CC: *B* = −6.273, *SE* = 2.459, *Wald* = 6.506, *P* < .011. The negative beta coefficients indicate that lower FA scores in these tracts increase the likelihood of an individual being preterm, and with increasing values of the FA in these tracts, there is a decreasing likelihood of an individual falling into the preterm group (Fig. 2B). The estimated probability plots from the final model can be found in the Supplementary Figure 1. Group comparisons reveal that FA in the l-ATR is significantly lower in the preterm group compared with the control group, controlling for the global FA value (*P* < .05, non-parametric one-way ANCOVA). For the FA in the l-UNC and R-CC, marginally significant group differences are observed (*P* < 0.0.07) with consistently lower FA values in the preterm cohort. Supplementary assessments for the RD, MD, and AD reveal significantly lower AD in the preterm group in all the identified tracts (L-ATR, l-UNC, and R-CC; Fig. 2B).

**Fig. 3.**
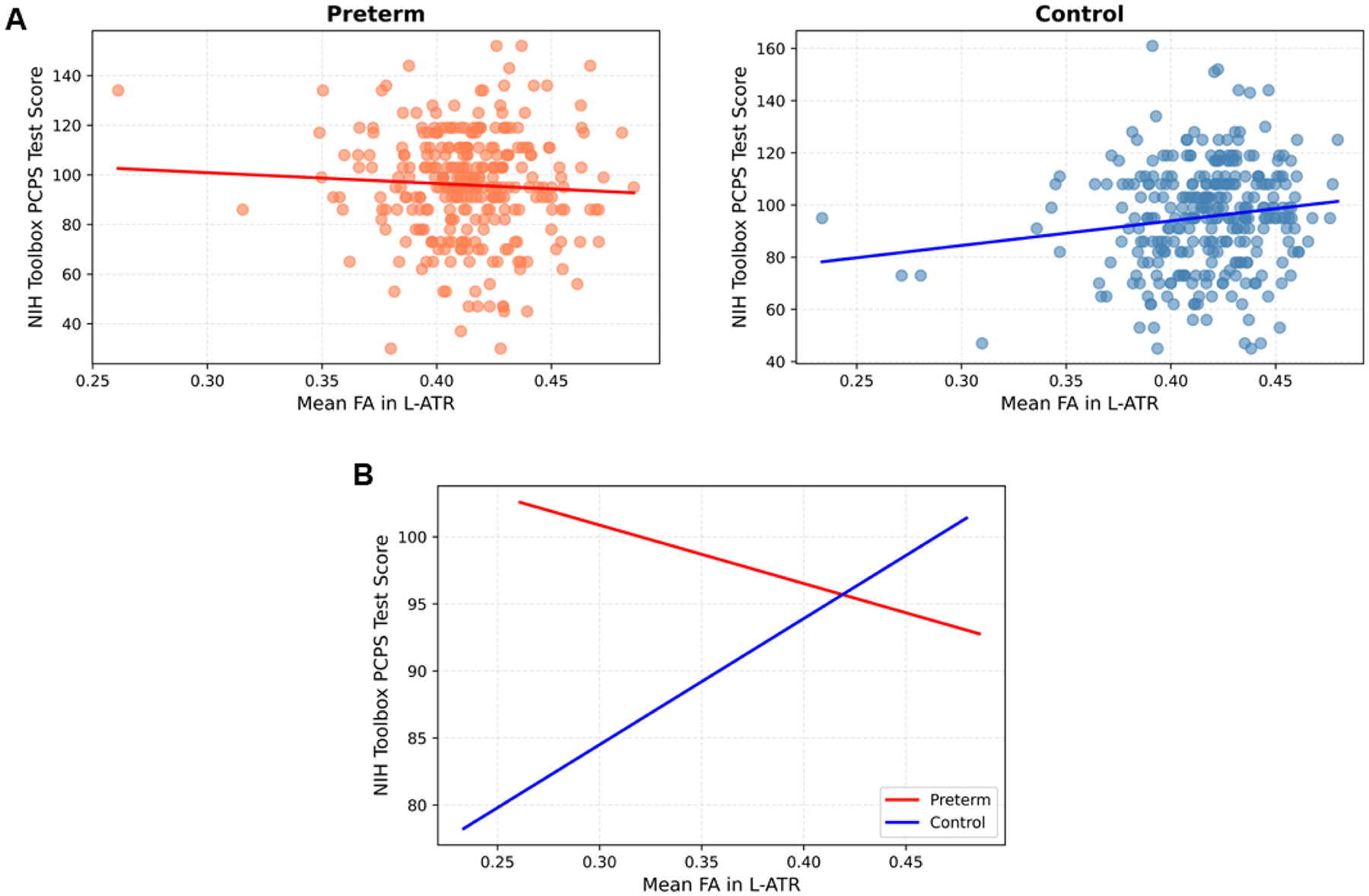
Altered brain-behavior regulatory white matter integrity in preterm children. (A) The control children show a significant low-degree positive relationship between the PCPS test performance and the FA in l-ATR. No positive brain-behavior relationship is found in the preterm children, who instead exhibit a tendency toward a negative relationship between these two measures. ** indicates *P*< .05 level significance. (B) The significant brain-behavior interaction indicates that the slopes of the FA–behavior correlations differ between groups. Specifically, the association between l-ATR FA and PCPS performance is significantly weaker in the preterm group than in the control group, reflecting distinct patterns of brain–behavior relationships.

Results of brain-behavior correlation analysis with the above-identified tracts show that the control group (and not the preterm group) shows a significant low-degree positive correlation between the performance on the Pattern Comparison Processing Speed (PCPS) Test and FA in the l-ATR (*r* = 0.15) and l-UNC (*r* = 0.17) (*P* < .05; Pearson’s correlations). No other correlation is found with other cognitive measures. No significant difference is found between the preterm and control groups for all cognitive measurements. Between-group assessments reveal a significant interaction effect of Group by Level of Performance of the PCPS test on the FA in l-ATR (and not in the UNC). In other words, the relationship (slope) between PCPS performance and the FA in l-ATR differs between the preterm and control groups (*P* < .05, non-parametric two-way ANCOVA controlling for the global FA; Fig. 3B), with the relationship in the preterm cohort being significantly weaker (with lower slope) than that in the control cohort. This brain-behavior interaction effect (relationship difference) is driven by the fact that the preterm children fail to show the normal-level positive relationship between the PCPS test performance and FA in l-ATR (Fig. 3A). The preterm group shows an opposite trend: a negative pattern of this neural-behavior relationship in the l-ATR.

## Discussion

The current study identified unconfounded neurobehavioral biomarkers that indicate childhood risks after premature birth. This was achieved by implementing a systematic confounding control approach, using the largest available dataset of brain development and child health (the Adolescent Brain and Cognitive Development Dataset). We found that non-medically complex preterm children are differentiated from the full-term control children by altered microstructural integrity in the l-ATR, l-UNC, and R-CC tracts in the whole brain, suggesting an underlying neuronal developmental pathway (detailed in discussions below). The identified tracts are major fronto-subcortical tracts that are important for communication between the primary (e.g., sensory, arousal, and attention) and the secondary (e.g., feedback, control, and decision making) cognitive processing. In these tracts, we found altered brain-behavior white matter microstructure, particularly implicating altered ATR integrity, in regulating the attention and processing speed performance in the preterm children. These results explain an increased risk of attention- and sensory-based dysfunctions, which frequently develop in preterm cohorts, thus providing new knowledge about the unconfounded neurobehavioral pathological processes of premature birth. The vulnerability of these specific frontal-subcortical connections—the Anterior Thalamic Radiation (ATR), Uncinate Fasciculus (UNC), and Corpus Callosum (CC)—in preterm birth could stem from the following aspects. The brain is particularly vulnerable to disruptions during critical phases such as neuromigration, differentiation, maturation, synaptogenesis, and the onset of myelination, which occurs during prematurity and could have lasting effects on neurodevelopment. (Kratimenos et al., 2025) Cerebral white matter axons (ie, projection, commissural, and association fibres) are in a phase of rapid growth during the premature period, the peak period of vulnerability for white matter injury. CC is reported as an important yet unique indicator of cerebral axonal disturbance in premature infants. The ATR has been reported to commonly affected by preterm birth and is especially correlated with infants with periventricular leukomalacia (PVL), a distinctive form of cerebral white matter injury. (Volpe, 2009) Preterm subjects were reported with lower FA values than term subjects in uncinate fasciculi, which showed significant positive correlations with later cognitive performance. (Mullen et al., 2011) The study further provides new clinical implications and suggests an urgent need for the development of more effective interventions and preventive efforts to forestall negative developments and enhance positive prospects for preterm populations.

### Frontal-subcortical neurosusceptibilities and axonal health in preterm children

Findings of the current study provide a new mechanistic understanding of the neurosusceptibilities following preterm birth unconfounded by postnatal and since-birth medical/developmental complications. First, children born preterm can be differentiated from the control children by altered microstructural connectivity in the l-ATR, l-UNC, and R-CC among all brain tracts. The deficiencies in FA and AD in these tracts in the preterm children reflect possible impaired axonal health in these brain regions. Fractional anisotropy (FA) indicates the strength of directionality of local white matter tracts and reflects axonal and myelin sheath integrity. (Beaulieu, 2002) AD measures the magnitude of the diffusivity parallel to the tract. (Pierpaoli and Basser, 1996) Decreased FA has been linked to impaired neuronal structure and axonal injury, (He and Parikh, 2015) while decreased AD has been linked to evidence of axonal damage. (Beaulieu, 2002; Hutchinson et al., 2018; Wheeler-Kingshott and Cercignani, 2009) Therefore, impaired FA combined with impaired AD identified in the preterm children in the current study can be attributed to possible axonal damage in these fronto-subcortical pathways, potentially associated with an abnormal process of synaptic pruning. (Beaulieu, 2002; Sowell et al., 1999)

Secondly, the ATR, UNC, and CC tracts consistently connect the anterior/dorsal (frontal) cortical regions and the subcortical structures. (Hwang et al., 2017) These neural pathways are important, serving to connect the primary processing (e.g., arousal, attention) and higher-order cognitive processing (e.g., decision making). Among these identified tracts, the ATR, which is most significantly implicated across all stringent statistical tests, primarily connects the dorsolateral prefrontal cortex with nuclei in the thalamus, (Alitto and Usrey, 2003) which is a unique brain structure that relays all sensory inputs to higher-order cortical areas for further processing and is critical to attention, arousal level, and alertness; the thalamus also plays a role in sensory-cognitive integration by mediating cortico-thalamo-cortical feedback and regulating cortico-cortical communications. (Hwang et al., 2017; Alitto and Usrey, 2003; Malekmohammadi et al., 2015; Sherman, 2016) The current result of altered ATR microstructural integrity and its underlying axonal health complements previous reports concerning the thalamic functionality in very preterm children. (Nunes et al., 2020) The current finding also provides the neural structural foundation that complements previous reports of diminished thalamic-cortical regulatory activity (Nunes et al., 2020; Ball et al., 2012; Cai et al., 2017) suggestive of deficient or slower neural maturation related to neural pruning and cortical maturation in preterm cohorts. (Ment et al., 2009; Mürner-Lavanchy et al., 2014)

Thirdly, in addition to the thalamus, the ATR fibers are also connected to the striatum regions, a neural system critical for motor control and reward feedback (Kamkar et al., 2017) and is therefore important for motor-related arousal control and monitoring. Lastly, the consistent identification of the cortical-subcortical tracts in the current results fills the gaps in previous studies that reported divergent neuropathological processes, subcortically in early life versus higher-order cortically in later childhood, in the preterm population. (Volpe, 2009; Shang et al., 2019)

### Dysregulated neurobehavioral neural structure in attention and processing-speed

We found a significantly reduced brain-behavior relationship in the preterm (compared to the control) children between the magnitude of brain connectivity (FA) in the frontal-subcortical connectome, particularly in the ATR, and the performance of attention and processing speed as captured by the PCPS Test. Processing speed is highly vulnerable to neuropathological changes and has been shown consistently to be the most sensitive neuropsychological measure for detecting underlying neurological deficits. (Carlozzi et al., 2015) The PCPS Test requires focused attention (alertness) and efficient attention-motor coordination, both of which reflect the ATR’s functionality involving the thalamus, in terms of its major role in relaying sensory stimuli for attention and further cognitive processing. Therefore, the current finding of dysregulated processing speed functionality characterized by the PCPS test implicated in the preterm cohort’s impaired ATR integrity provides further evidence of possible neural damage consistent with the earlier findings of impaired diffusivity (FA and AD) in the ATR neurocircuit, suggestive of an underlying neuronal deficit (i.e., in the axons).

### An increased risk of attention- and sensory-related dysfunctions in preterm children

While the preterm cohort shows normal levels of cognitive performance in behavioral measures, they failed to show the normal-level brain-behavior regulatory ability in the ATR linked to attention and processing speed. The reduced brain-behavior regulatory capacity in preterm children suggests impaired neural structure and activity in behavioral development. This impairment may leave the preterm children more susceptible to failing at tasks that require more attention and cognitive speed when the non-specialized, dysregulated brain can no longer meet increasing environmental demands. Previous meta-analysis has generally suggested that children born preterm are at greater risk for reduced cognitive test scores. (Bhutta et al., 2002) Limited preterm studies that reported neurocognitive and behavioral outcomes have shown a general pattern of subtle and non-specific deficits associated with premature birth, with a trend for worsening outcomes over time attributable to increased environmental demands. (Aylward, 2002a; Taylor et al., 2000)

Results of the current study provide an etiological explanation of the cognitive processes underlying certain neurodevelopmental dysfunctions highly developed in preterm populations. The altered brain-behavior regulatory neural structure indicates an increased risk in the preterm children for developing attention- and processing speed-related behavioral and mental dysfunctions. Prior research often reported certain neurodevelopmental outcomes frequently developed in children born preterm, particularly including attention deficit/hyperactivity disorders (ADHD), neuropsychological dysfunctions (e.g., related to visuo-motor coordination), learning disabilities, and borderline intellectual disabilities. (Bhutta et al., 2002; Allotey et al., 2018; Sucksdorff et al., 2015; Aylward, 2002a) In particular, children born preterm exhibit the highest likelihood of developing ADHD, (Bhutta et al., 2002; Allotey et al., 2018; Sucksdorff et al., 2015; Aylward, 2002a, 2002b) a disorder that is associated with frontolimbic (i.e., frontostriatal and cingulate) dysfunctions. (Bush, 2011) All of these widely reported incidents in preterm survivors commonly implicate shared features of dysfunctionality in attention, sensory-motor integration, and response coordination. Findings of the current study provide an etiological explanation suggesting that impaired microstructural integrity in the fronto-thalamic-striatal circuits in preterm children, linked to altered attention and processing speed regulation, may underlie the high incidence of related disorders such as ADHD, (Allotey et al., 2018; Sucksdorff et al., 2015) sensory-motor dysfunctions, and learning/intellectual disabilities frequently developed in preterm children. Meanwhile, although the preterm group exhibited no significant differences in cognitive performance compared with the term-born controls, they showed altered microstructures in key white matter pathways such as the ATR and uncinate fasciculus. This pattern may suggest that atypical white matter organization in preterm children reflects adaptive or compensatory neurodevelopment. Specifically, the altered white matter integrity in the preterm group could indicate that the preterm brain reorganizes its neural architecture to support behavioral functions comparable to those of term-born peers—suggesting potential greater neural plasticity. However, longitudinal studies tracking both microstructural changes and cognitive outcomes over time are needed to disentangle the neuroplasticity or the compensatory reorganization from delayed maturation in the preterm brain. These neurobehavioral susceptibilities may leave these children at greater risk of later developing related behavioral, neurocognitive, and mental dysfunctions when environmental demands increase.

### An urgent need for effective targeted interventions for preterm populations

The findings of this study suggest a critical need for more specific and targeted interventions that can forestall negative prospects for preterm populations. Such interventions are currently lacking in the field, which results in many preterm-born individuals suffering silently and struggling unnecessarily. Neurobehavioral rehabilitative training programs that target specific neurocognitive susceptibilities, including sensory-feedback, attentional training, and visual-motor coordination, combined with speed training, are applicable and beneficial, and may effectively reduce the risk of these children developing related neuropsychological dysfunctions. Furthermore, insufficient attention has been paid to seemingly healthy preterm children at birth (i.e., born without extreme postnatal complications), and little effort has been made to follow these newborns into their childhood. The findings of this study suggest a need for specific interventions that can prevent negative developments for these at-risk children. Developmental surveillance through regular follow-ups of the preterm children, which has not been routinely done before, may be necessary to identify early dysfunctions at the soonest. This will make it possible to implement early ameliorative interventions that ensure long-term prospects for these children.

### Limitations and future directions

In this study, we did not limit the preterm cohort’s eligibility based on the gestational age, or statistically control for it, because we sought to identify key findings generalizable to all premature ranges of preterm populations. Additional questions regarding how the level of prematurity at birth may influence the identified results can be addressed in further studies. In addition, future investigations are needed to follow up the clinical development of neurocognitive impairment and mental health conditions in these at-risk preterm children to further disentangle the mechanism of dysregulation/impairment or neural reorganization from compensatory pathways for the preterm brain.

## Conclusions

Using a systematic quality control approach, this study resolved critical methodological issues not addressed in prior preterm research, and revealed unconfounded childhood impact of premature birth and its underlying neurobiological and cognitive basis. Preterm children exhibit altered white matter development and brain–behavior relationships, which may leave them susceptible to varied and increased environmental demands requiring attention and cognitive speed. Findings of this study shed new light on the possible development of effective and targeted interventions, as well as preventive measures, for preterm populations.

## Supplementary Material

1

Supplementary material associated with this article can be found, in the online version, at doi:10.1016/j.neuroimage.2025.121600.

## Figures and Tables

**Table 1:** Demographic information of the study-eligible children for analysis. Note: The control group and the preterm group are matched on each demographic variable, including gender and handedness (number match) and age and socioeconomic status (SES) (statistical match).

	Age at scan/assessment (years)	Gender (number of girls/boys)	Handedness (number of right/left/ ambidextrous)	SES: Primary Care Giver Education (years)	SES: Total Family Annual Income (Level)	Gestational Age At Birth (weeks)
**Preterm Children** (*N* = 306)	9.92±0.60	140/166	248/21/37	16.59±2.56	7.49±2.11	34.94±2.38
**Control Children** (*N* = 306)	10.03±0.55	140/166	248/21/37	16.45±2.73	7.46±2.15	N/A
**Independent T-test P value**	*P*=.11			*P*=.73	*P*=.87	

## Data Availability

Data collected for this study are sourced by the public available, ABCD Study dataset, available for downloaded from: https://abcdstudy.org/scientists/data-sharing/. Additional information and the source code of the model are available with publication upon request to the corresponding author.
