# Supplementary material for: Altered neurobehavioral white matter integrity in preterm children: A confounding-controlled analysis using the adolescent brain and cognitive development (ABCD) study
Source: Neuroimage. Author manuscript; Available in PMC 2025 Dec 18. (PMC12712894; doi:10.1016/j.neuroimage.2025.121600)
Supplement: 1 [file NIHMS2127587-supplement-1.docx]

**Altered Neurobehavioral White Matter Integrity in Preterm Children:**

**A Confounding-Controlled Analysis Using the Adolescent Brain and Cognitive Development (ABCD) Study**

**Supplementary Methods**

***Participant inclusion/exclusion and quality control procedure***

To identify eligible study groups unconfounded by medical and developmental history since birth, the following quality control assessment procedure was carried out (Figure 1A). We first excluded participants that met *any* DSM-V diagnosis of psychiatric disorders since birth using the Kiddie Schedule for Affective Disorders and Schizophrenia (KSAD-5 full parent report; Orvaschel 1994). Next, we excluded children with postnatal complications, including: blue at birth, had slow heartbeat, had convulsions, did not breathe at first, required oxygen, blood transfusion, or incubator after birth, had Rhesus incompatibility, had any infections or serious illness within the first 12 months of life (excluded N=1279; using the [*ABCD Developmental History Questionnaire*](http://nda.nih.gov/data_structure.html?short_name=dhx01), parent-report). Furthermore, we examined head trauma and neurological history and excluded children with a medical history of: head related injury (including traumatic brain injury, head/neck injury, being knocked unconscious), any neurological conditions (including cerebral palsy, epilepsy, seizures, or multiple sclerosis), severe headaches, or hearing or color vision problems (excluded N=613; using the [*ABCD Parent Report Medical History Questionnaire*](https://nda.nih.gov/data_structure.html?short_name=abcd_mx01) and [*ABCD Parent Report Ohio State Traumatic Brain Injury Screen Questionnaire*](https://nda.nih.gov/data_structure.html?short_name=abcd_otbi01), modified short form). This results in a total of 2268 demographic-eligible healthy children for the current study who are free from medical, neurological, and mental health confounding conditions since birth, including 417 children born preterm and 1851 full-term born children. The preterm/full-term criteria were based on the *ABCD Parent-Report Medical History Questionnaire*. Sociodemographic factors, including age, gender, handedness, and socioeconomic status (determined by the primary care giver's highest education level, and the total household annual income) (Braveman 2005) were recorded from the [*ABCD Parent Demographics Survey*](https://nda.nih.gov/data_structure.html?short_name=pdem02).

***Neuroimaging and quality control process***

The imaging data of the above demographic-eligible children were assessed with the following quality control procedures to identify eligible data for the imaging analysis for the current study. We excluded participants with incomplete imaging records, with abnormal MR findings, data that failed raw image quality checks (QC) and post-processing QC, including data that failed QC for raw T1 and diffusion MR image series, with insufficient fMRI total number of repetitions for all OK scans, with unavailable scans with B0 unwarp; or data that failed FreeSurfer post-processing QC, image registration (dMRI to T1), or brain coverage QC (dorsal and ventral cut-off checks) (excluded N=542) (Figure 1B). A final eligible Preterm group (N=306) was identified, and a matched Control group (N=306) was obtained from the total eligible control pool using a randomization procedure to match the Preterm sample, including exact match in number, gender, and handedness, and statistical match in age and socioeconomic status (Table 1). In all data, high resolution anatomical (T1-weighted) and diffusion MR images were acquired for each participant. Harmonized multi-center imaging scan parameters, detailed in Casey et al. (2018), were used across sites (e.g., MRI: TR=2500ms; flip angle = 8 degrees; matrix=256x256; resolution=1.0mm isotropic; ). The dMRI was acquired using multiband EPI [1, 2] with slice acceleration factor 3 and includes 96 diffusion directions, seven b = 0 frames, and four b-values (6 directions with b = 500 s/mm2, 15 directions with b = 1000 s/mm2, 15 directions with b = 2000 s/mm2, and 60 directions with b = 3000 s/mm2). [3] DTI data and derivatives used in this study were obtained from the ABCD Study, which provides minimally processed dMRI derivatives. The standard ABCD processing [3] includes eddy current distortion correction, $B_{0}$ distortion correction, registration to structural MRI, tensor fitting and modeling, fiber tract segmentation, and automated QC. The automated post-processing quality control (QC) metrics were statistically characterized in association with manual QC variables. The statistical association between manual and automated QC variables was used to guide the identification of automated QC variables that were predictive of manual QC scores. The pairwise analysis of manual-vs-auto QC scores lead to automated QC variable thresholds needed to identify anomalous scans with desired false alarm ratings of 0.05%. Finally, the anatomical T1 and DTI scan series of the final study eligible data were processed manually based on the ABCD recommended thresholds. Further detailed decisions and processing for how the ABCD Study data are recommended for inclusion in imaging data analysis can be found in Casey et al (2018).

These diffusion measures were extracted from a total of 30 white matter regions consisting of 15 major tracts throughout the whole brain, which include: (1) subcortical/subcortical-cortical tracts: fornix, bilateral cingulate-cingulum, parahippocampal cingulum, corticospinal tract, anterior thalamic radiation, uncinate fasciculus; (2) cortico-cortical tracts: anterior and posterior forceps, bilateral inferior longitudinal fasciculus, temporal and parietal superior longitudinal fasciculus, inferior-fronto-occipital fasciculus, superior corticostriatal tract, superior corticostriate-frontal and -parietal white matter. A global FA value (mean FA value of the whole brain) is used for each participant as a covariate to control for individual baseline connectivity differences in all analyses.

***Neurocognitive Measurement***

We use the following NIH Toolbox cognitive tests spanning the full-range information processing functionality. (1) *NIH Toolbox Pattern Comparison Processing Speed*: This test measures attention and information processing speed, by asking the participants to look at two pictures at a time and respond yes or no as fast as they can with button presses to indicate whether the two pictures are identical or not. (2) *NIH Toolbox Flanker Inhibitory Control And Attention Test*: This test measures inhibition and cognitive control (executive function) by asking the participant to respond to the direction of the target arrow while ignoring (suppressing) distracting arrows that point to the same or different direction. (3) *NIH Toolbox Dimensional Change Card Sort Test*: This test measures cognitive flexibility (executive function) by asking the participant to match either the shape or the color between the target and the selected items based on the matching rule that changes. (4) *NIH Toolbox List Sorting Working Memory Test*: This test measures visual and auditory working memory capacity, which requires the participant to remember a series of items (seen and heard) and respond verbally by the order of size (from smallest to the biggest). (5) *NIH Toolbox Picture Vocabulary Test*: This test measures language ability and vocabulary knowledge by asking the participants to choose the best item from several pictures they see that matches the meaning of each word they hear. (6) NIH Toolbox Picture Sequence Memory Test: This test measures episodic memory by asking the participants to sort the sequences of the pictures in the original order as presented to them. (7) NIH Toolbox Oral Reading Recognition – measuring reading decoding skills and crystalized abilities. Raw scores for each test were obtained and converted to the age-adjusted standard scores, which (the latter) were used for all analyses

**Supplementary Figures**


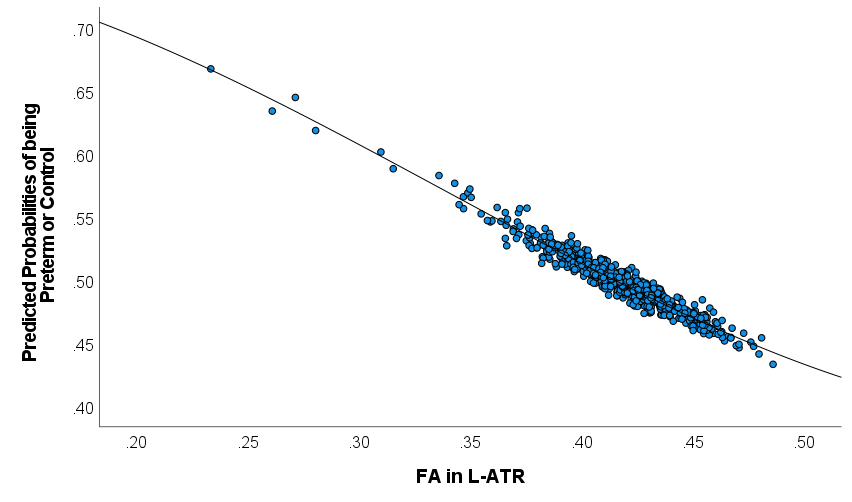


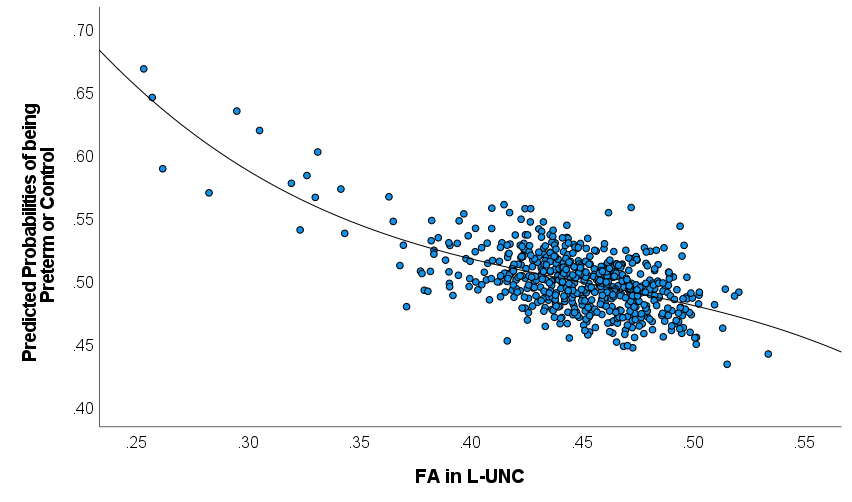


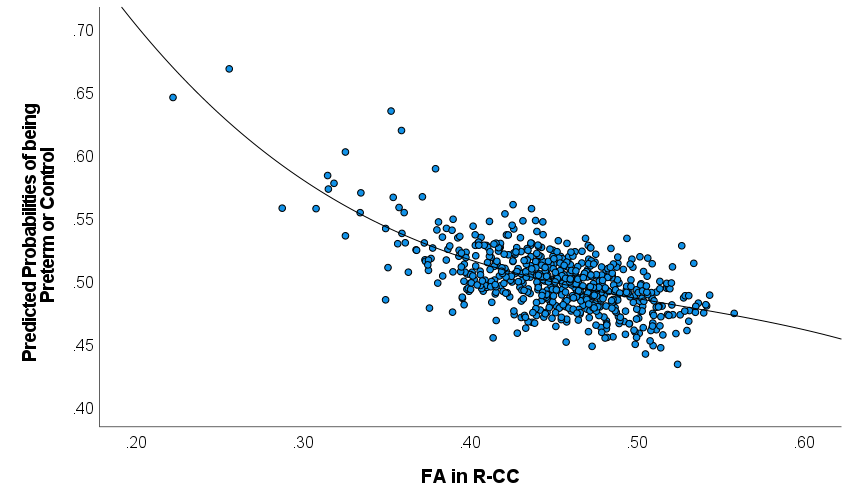


**Supplementary Figure 1**. Predicted probability plots for each identified significant white matter tract. The predicted probabilities (Y-axis) of a child being a preterm or control (values ranging from 1 to 0) are plotted against the FA in each identified tract (X-axis). For visualization purposes, a cubic line is fitted in for each tract to show the predictive relationship.

1. Moeller, S., et al., *Multiband multislice GE-EPI at 7 tesla, with 16-fold acceleration using partial parallel imaging with application to high spatial and temporal whole-brain fMRI.* Magn Reson Med, 2010. **63**(5): p. 1144-53.

2. Setsompop, K., et al., *Blipped-controlled aliasing in parallel imaging for simultaneous multislice echo planar imaging with reduced g-factor penalty.* Magn Reson Med, 2012. **67**(5): p. 1210-24.

3. Hagler, D.J., Jr., et al., *Image processing and analysis methods for the Adolescent Brain Cognitive Development Study.* Neuroimage, 2019. **202**: p. 116091.
